# A qualitative study of the views of healthcare professionals on providing vaccines information to patients

**DOI:** 10.1007/s11096-021-01299-y

**Published:** 2021-06-21

**Authors:** Ruth Loftus, Laura J. Sahm, Aoife Fleming

**Affiliations:** 1 Pharmaceutical Care Research Group, School of Pharmacy, University College Cork, Cork, Ireland; 2 Pharmacy Department, Mercy University Hospital, Grenville Place, Cork, Ireland

**Keywords:** Healthcare professional, Pharmacist, Qualitative, Vaccination, Vaccine hesitancy

## Abstract

*Background* Healthcare professionals (HCPs) such as pharmacists, general practitioners and practice nurses are a trusted source of vaccines information for patients in primary care. Global regulators have highlighted the key role of HCPs in fostering confidence in COVID-19 vaccines. *Objective* This study aims to gain insight into the views and experiences of HCPs on providing vaccines information to patients. *Setting* Primary care general practice surgeries and community pharmacies in Ireland. *Methods* Qualitative, semi-structured interviews were conducted with 14 HCPs (five General practitioners [GPs], four practice nurses and five community pharmacists) identified through purposive and convenience sampling. The interviews were analysed by inductive thematic analysis. *Main outcome measure* Participants’ views and experiences of providing vaccines information to patients. *Results* Five key themes were identified: roles and responsibilities, perception of risk, perception of the public, building a relationship, and emotion. HCPs were motivated by duty and care for their patients. They respected patient autonomy and were driven by their concern for public health. HCPs were influenced by their perception of risk and their perceptions of the public. HCPs practiced patient-centred care by providing tailored vaccines information. They favoured an approach of providing patients with information and support to make their own decision. The topic was emotive; HCPs empathised with patients but were also frustrated by their perceived inability to change some patients’ views. *Conclusion* The provision of vaccines information by HCPs to patients is multifactorial with participants mindful of patient autonomy and the HCP role to support vaccinations as a public health priority. Participants suggested that education and support on vaccines communication would enable them to support the vaccines uptake in their practice.

**Supplementary Information:**

The online version contains supplementary material available at 10.1007/s11096-021-01299-y.

## Impacts on practice

- Healthcare professionals are mindful of the importance of patient autonomy, the importance of a trusting HCP-patient relationship, and understanding patients’ perception of risk, when patients make decisions on vaccination.
- Healthcare professional educational and continuing professional development bodies may need to consider HCP training and education to support the consistent, multidisciplinary communication of vaccines information, to support vaccines uptake.

## Introduction

The success of vaccines as a lifesaving and cost-effective medical intervention is well established. At least ten million deaths are estimated to have been prevented by vaccines between 2010 and 2015 [1]. In 2019, however, Albania, Czechia, Greece and the United Kingdom lost their measles-free status, forcing the WHO to declare an emergency response [2]. This concerning trend highlights the importance of maintaining immunisation uptake rates to reduce the transmission of vaccine preventable diseases (VPD).

The WHO *Measuring Behavioural and Social Drivers of Vaccination* group highlights the importance of the provider recommendation in vaccine decision making, supported by recent publications [3–7]. Global regulators have highlighted the key role of Healthcare Professionals (HCPs) in fostering confidence in COVID-19 vaccines [8]. It has been recognised that HCPs need support when talking to patients and the public on COVID-19 vaccines to ensure that patients are reassured of the safety of the vaccines and the robustness of regulatory processes. *Harmsen *et al*.* found that information seeking behaviours vary among parents regarding childhood vaccinations in results of an online questionnaire of parents, with some parents not receiving enough information on side effects [9]. Insight into the type of information sought by parents, and whom they seek more information from is important to consider when developing vaccine communication resources for the public and HCPs. A recent study found that vaccination uptake correlated positively with those who valued information provided by HCPs, in contrast to others who preferred information sources such as social media and friends, in which case vaccine uptake was negatively affected [10]. Research shows that HCPs maintain strong loyalty to practice guidelines and feel a duty to recommend vaccines, especially scheduled vaccines [11–13]. The National Institute of Health and Care Excellence (NICE) [14] guidance recommends that further research is needed to identify the most effective ways to increase vaccine uptake, for example, through changes in information provision and the introduction of opportunities to discuss immunisation before vaccines are given [15].

HCP performance in this role is influenced by variables including their own knowledge of, and confidence in a vaccine [16, 17], the availability of evidence-based information [13, 17], and opportunity to promote vaccines. Studies have shown that vaccines are not always proactively discussed by HCPs with patients [12, 18, 19] and environmental constraints such as time and the priority of other medical issues are significant barriers [18–21]. Leask et al*.* developed a framework for communicating with parents about vaccination and the recommendations are tailored to specific parental positions on vaccination, primarily based upon building rapport and respectful interactions [7]. This tailored approach to communicating with patients and responding to their information seeking behaviour is recommended, but largely not implemented in practice. A review of qualitative studies exploring the attitudes of HCPs surrounding vaccinations found that most HCPs regarded vaccines positively and felt a duty to recommend them [17, 18, 22–34]. The role HCPs play in providing accurate information and advocating for vaccination is crucial and requires further investigation.

### Aim of the study

This study aimed to examine HCP views and experiences on providing vaccines information to patients in their practice.

### Ethics approval

Ethics approval for the study was granted by the Social Research and Ethics Committee at University College Cork (on 07/03/2019). (Log 2019-015). Participants provided written informed consent.

## Methods

A qualitative, semi-structured interview study was conducted to obtain in-depth, detailed accounts of participant’s experiences, perspectives and opinions [35]. The topic guide was developed based on the research teams prior research in the area and it was piloted with a community pharmacist and minor changes were made.

The participants, and their practice setting and role in vaccination, included:

- Community pharmacists (working in a retail pharmacy business, administer and dispense vaccinations).
- General practitioners (GPs) (family doctor/physician, prescribe and administer vaccinations).
- Practice nurses (working in a primary care GP practice setting, administer vaccinations).

HCPs were recruited using purposive and convenience sampling from primary care practices and pharmacies in the West of Ireland [36]. An initial analysis sample of ten and a stopping criterion of three was agreed by the authors was used to determine when data saturation had been reached [37].

### Data collection

HCPs were first contacted by telephone and subsequent letter detailing the purpose of the study. The participants were aware that the interviewer was a pharmacist and researcher. Semi-structured interviews were conducted face to face by RL (a community pharmacist and researcher), between April 2019 and August 2019. The audio recordings were transcribed verbatim by RL.

### Data analysis

The codes were analysed using inductive thematic analysis and the constant comparison approach as per Braun and Clarke [38]. On the basis of the initial and iterative familiarisation of the transcripts by all authors it was decided not to analyse the transcripts in three separate HCP groups. This decision was supported by the use of the same topic guide for all HCP groups, and similar issues were reported across the HCP groups. All transcripts were coded independently by RL and one other author (AF or LJS). The resulting generated themes were discussed and reviewed collectively, and the final set of themes was defined by consensus. Representative participant quotations are presented to illustrate each theme and subtheme in the results [38]. Reporting was guided by the Consolidated Criteria for Reporting Qualitative Studies (COREQ) checklist [39] (see Supplementary material).

## Results

### Participant information

In total, 14 participants (6 female) were interviewed, consisting of five community pharmacists, five GPs and four practice nurses all with a range of years of experience. Mean interview duration was 28 min (range 19–66 min). Thirteen out of 14 interviews were audio recorded; one participant declined to be audio-recorded, detailed notes were taken throughout.

### Predominant themes

Five themes emerged from the data (Table 1): Perception of risk, Roles and responsibilities, Perceptions of the public, Building a relationship and Emotion.

**Table 1:** Themes and sub-themes and their occurrence in each interview

Theme	Participants													
Subtheme	P1	P2	P3	P4	P5	P6	P7	P8	P9	P10	P11	P12	P13	P14
*Roles and responsibilities*														
Risk vs benefit	✓	✓	✓	✓	✓	✓	✓	✓	✓	✓	✓	✓	✓	✓
Risk to public health	✓	✓	✓	✓	✓	✓	✓	✓	✓	✓			✓	✓
Confidence in evidence	✓	✓	✓	✓	✓	✓	✓	✓	✓	✓	✓	✓	✓	✓
*Perception of risk*														
Education	✓	✓	✓	✓	✓	✓	✓		✓	✓	✓	✓		✓
Represent public health	✓	✓	✓	✓	✓	✓	✓	✓	✓	✓	✓	✓	✓	✓
Role models			✓		✓		✓	✓	✓			✓		
Potential to do more	✓	✓	✓	✓				✓	✓	✓	✓		✓	✓
*Perception of the public*														
Lack of knowledge		✓	✓	✓	✓	✓	✓	✓	✓	✓	✓		✓	✓
Outside influences	✓	✓	✓	✓	✓	✓	✓	✓	✓	✓	✓		✓	✓
Preconceived views	✓	✓	✓		✓	✓	✓	✓	✓	✓	✓	✓	✓	✓
Protect individual vs public		✓				✓	✓	✓		✓		✓		✓
*Building a relationship*														
Patient-centered care	✓	✓	✓	✓	✓	✓	✓		✓	✓	✓	✓		✓
Respect patient autonomy	✓	✓	✓	✓	✓	✓	✓		✓	✓	✓	✓	✓	✓
Trust		✓	✓	✓			✓			✓	✓	✓	✓	
*Emotion*														
E.g., frustration, concern, empathy		✓	✓		✓		✓	✓	✓	✓	✓		✓	✓

### Roles and responsibilities

#### Education

HCPs identified their primary role in providing immunisation services as being educators. They spoke of their responsibility to dispel myths, diffuse misinformation, and provide accurate information regarding risks and benefits to enable the patient to make an informed decision.

I provide education and information…I explain what the vaccine is, what is in it. I explain the disease it is preventing, and I go through all the risks and side effects that can happen so they know what to expect and they can make an informed decision – P12 – Practice Nurse (PN)

#### Represent public health

HCPs felt they acted as conduits to disseminate best practice guidance, for which they looked to national bodies, primarily the Health Service Executive (HSE) or in some cases the National Health Service (NHS) in the United Kingdom [14] or Centre for Disease Control (CDC) in the United States of America (USA). HCPs expressed faith in the national immunisation schedule and believed it is their job to convey its recommendations to patients and/or to be knowledgeable if asked. While they are provided communications by the HSE, or their professional organisation, they believed there was an onus on them to continue their professional education to reliably carry out their vaccines advisory role.

The HSE is advising in the situation, we have a national programme that works. So, it’s not just me as an individual telling them to get vaccinated, this is the programme, this is what we have, it’s respected all over the country, you know there’s a good base for it. – P13 – GP

#### Role models

HCPs felt that they have a responsibility to serve as role models. They believed that to promote a vaccine, they must believe in it themselves, and practice what they preach.

Usually I say, ‘I have two children, they’ve had the vaccines,’ and that kind of reassures people...Patients are more reassured when they hear it in real terms. It’s not just quoting stats at them, they think ‘she’s giving it to her child, it must be okay,’ rather than me saying one thing and doing another. - P5 – PN

Participants believed that information disseminated from HCPs should be consistent, and that there was a danger that variable information from HCPs could hamper vaccination uptake, and potentially weaken the public’s trust in the medical community.

People aren’t quite trusting of HCPs because you can get differing opinions… If you get a diagnosis, you might get one opinion from one person, and a different one from another person… it might diminish their trust in HCPs - P3 – PH (Pharmacist)

While all participants accepted their role in providing information, some conceded that they had the potential to expand this role, as there was often the assumption that the information was provided by another HCP in a different setting.

We assume that the GP, or whoever is giving the vaccine, will educate the patients. It’s only when people come into us and they ask us questions…then we have to go off and educate ourselves - P3 – PH

### Perception of risk

#### Risk versus benefit

In the context of vaccines, HCPs considered the risk versus benefit ratio. All participants expressed the belief that the risk of disease far outweighed the risks associated with the vaccine. HCPs spoke of their own experiences with VPDs as a powerful argument to support this perception. Faith in evidence and statistics was frequently reported.

It speaks for itself, all of the statistics, the drop in childhood diseases like polio, mumps measles, and sadly that is going up at the moment. I have two children and they have had all their vaccines…I just think, if I can stop them being sick, I’m going to do that, because to me, the risk of being sick is far more than any tiny risk…with the HPV one, there’s a whole lot of scaremongering with this chronic fatigue thing, but to me, I’d rather my child potentially have that, than cervical cancer, but a lot of it is based on facts and statistics and my background. I’ve seen the other side of it, I’ve seen a child with mumps and measles. - P5 – PH

#### Risk to public health

HCPs not only acknowledged the risk of the individual contracting the VPD but spoke of the risks posed to the greater community by those who refuse vaccination.

The more people that are immune to the flu, the less chance it has of spreading, and that’s what herd immunity is for a lot of the other stuff that we almost eradicate. - P3 – PH

#### Confidence in evidence

Participants agreed that vaccines are not without risks and were realistic about the possibility of associated side effects or long-term adverse effects but expressed confidence in experience and in the pharmaceutical process. They therefore perceived these risks as being minor and rare. Some participants expressed hesitancy when newer vaccines are added to the immunisation schedule, and two spoke of uncertainty surrounding the safety of antenatal and new childhood vaccines. In these instances, the HCPs perceived the risks of the vaccine as being possibly greater than the benefits due to lack of experience or evidence to suggest otherwise.

The men. B was a huge one for us. Firstly, nobody knew what to make of it; we were all worried…because it came with this thing about the temperature and unknown side effects. I remember saying to the GP, ‘I hope they don’t go ahead with this Men. B’ because it just sounded really scary…. but now, I think it’s great, there were way too many fatalities [without it], and they were preventable - P9 – PN

Other HCPs felt confident regarding the risk–benefit profile of vaccines in pregnancy due to ongoing experience suggesting an absence of adverse effects and a reduction in the prevalence of disease.

I promote the pertussis vaccine in pregnancy, I’m very for it. I know a lot of GPs that aren’t, which surprised me a little bit at the start… There has been very few, or no adverse effects that I have heard anyway, since we have started. When it comes to the foetus, I have no problem with it, I’m quite comfortable. Some GPs don’t like it, because they think it is just an intuitive thing that you don’t give anything in pregnancy if you don’t have to, but with the numbers of pertussis deaths, I think the benefits outweigh the risks - P14 – GP

### Perception of the public

#### Lack of knowledge

Participants knew their patients often perceived vaccines differently to them. Consequently, they attempted to empathise; to view and understand the subject from the public’s perspective. HCPs believed that much of the mistrust or scepticism around vaccines stemmed from fear of the associated pain of injection, or fear of the unknown, lack of knowledge or misinformation.

Let them make an informed decision and a lot of times they will come back, because the reason they weren’t getting it in the first place was they were afraid of it - P4 – PH

Some HCPs suggested that the success of immunisation schedules in reducing the incidence of VPDs could have led to public complacency and a perceived low of risk of contracting the VPD, thus reducing vaccines uptake. It was commonly reported that often when the patient was fearful of the disease, they were willing to pay for a vaccine privately, despite cost being a barrier to vaccination in many instances. Other times, participants believed patients were more fearful of the risk of side effects than the risk of contracting the disease.

The thing about men. B is I think people have a good understanding of the severity of the illness and that’s a factor, so one of the problems with the MMR now, is that people don’t realise how nasty a condition the measles is, the MMR now has become a victim of its own success – P11 – GP

#### Outside influences

While participants expressed confidence in vaccines due to supporting evidence, they believed that the public were more influenced by friends or relatives, the media, sensational stories, and anecdotes; all of which can drive the patient towards, or against vaccination.

We [HCPs] are more inclined just to believe science, facts and figures. There’s research behind all these things, but other people don’t have that same background and maybe we don’t appreciate that. People don’t see things the way we do – P3 – PH

#### Preconceived views

Participants believed that patients usually had preconceived views about a vaccine before speaking to them. They felt some patients thoroughly researched their views, and this led them to either a pro or an anti-vaccine sentiment. In the latter case, HCPs perceived that it was difficult to change that patient’s mind.

There will be people who have done their own research and have read up on it, and they will absolutely refuse because they feel something will come to light down the line. They can’t be changed – P6 – GP

In other cases, HCPs felt that accurate information gave most people reassurance and confidence to proceed with vaccination. They believed that most people who seek information about vaccines were open to the possibility of immunisation.

If you dispel some of the myths…once you go through the information and explain it…then I think most people accept it - P14 – GP

#### Protect individual versus public

HCPs felt that the primary motivator behind a person’s decision to vaccinate was to protect themselves or their children. While HCPs spoke of their own desire to protect the health of the wider community, they did not feel that herd immunity was a concept that the public understood or, to which they were receptive.

People look after themselves and their own family first. They don’t care as much about the public health element of it. We might find that difficult to understand because we’re always thinking about public health – P14 – GP

### Building a relationship

#### Patient centred care

Many participants expressed that they get to know their patients very well and practised patient-centred care. They recognised the importance of spending time with their patients to hear, and address, concerns surrounding vaccines. They reported including the patient in the discussion and speaking to them without using medical jargon.

I’m trying to work with them, I’m not trying to say, ‘vaccines are brilliant and there’s nothing that could possibly go wrong with them.’ It’s about balancing risks and benefits, and I’m trying to develop a level of trust and credibility by saying ‘yes, I understand your concerns' - P 11-GP

#### Respect patient autonomy

Participants believed that it was important to respect the patient’s autonomy, despite possibly not agreeing with their beliefs. They try not to come across as forceful or paternalistic regarding the subject of vaccines. They also spoke of the importance of reassuring the patient and giving them the tools to take the responsibility for their decision.

If you tell somebody to do anything, it generates this sense of resistance. They resist that, whereas, if you transfer the responsibility for making the decision, they are a bit more likely to run with it – P11 – GP

#### Trust

Participants felt that when a patient began to trust them, the patient would ask for their opinion, which created an opportunity to educate. It was acknowledged that building a relationship with patients in this way took time, but that patients were then more likely to listen. This also facilitated participants in identifying and recommending the appropriate vaccines for patients throughout their lives.

I say ‘I would get the vaccine, I would avail of any opportunity to get my kids vaccinated’ and I think that is the best endorsement I can give…and they listen because they trust me – P12 – PN

#### Emotion

The subject of vaccines generated emotion in participants. HCPs showed empathy towards patients who expressed fear or challenges as reasons against getting vaccinated.

We have a couple of families who have a child with autism and won’t give their second child the MMR just in case… It really doesn’t matter how much you’re going through it with them that there is absolutely no correlation between the two… I can sort of see their point; they’re scared of it! – P9 – PN

In other cases, HCPs demonstrated frustration when patients resisted their advice and appeared to place more trust in what HCPs perceived to be misinformation, sometimes from other HCPs. Participants felt helpless to change some patients’ views.

She heard from a GP under no circumstances to get the whooping cough vaccine while pregnant, because it causes serious defects in the baby, so she was absolutely petrified… It was total misinformation, I was horrified – P9 – PN

Several pharmacists noted that they were less likely to come in contact with patients who were vaccine hesitant because *“somebody who does not believe in vaccines is just not going to raise the question” (P1-Ph).* A practice nurse reinforced the importance of building a trusting relationship with a patient who may be vaccine hesitant, and that by recounting her own personal experience of vaccinating her children this helps some parents to accept vaccination. Most of the HCPs in the study believed that providing reassurance and evidence-based information to hesitant patients was the approach they take.

HCPs exhibited concern and regret for the individual and the wider community when patients remained unwilling to vaccinate, despite being informed of its public health benefits. They feared the potential spread of VPDs they feel could be prevented and eradicated.

you can’t really argue 100% that vaccines can’t cause trouble… their children’s health is piggybacking on the goodness of everyone else who has been vaccinated, if it wasn’t for the people that are vaccinated, they would have a higher disease burden – P8 – GP

### Different HCP views

All participants viewed vaccines positively and recognised their role in the delivery of immunisation services. Community pharmacists in this study were more familiar with the influenza vaccine. In Ireland Community pharmacists now provide around 10% of total influenza vaccines in Ireland [40]. Pharmacists reporting feeling less confident providing information relating to other specific vaccines on the immunisation schedule but believed that they were important to promote. Except for the influenza vaccine, they believed that most information provision occurred in the GP surgery. The pharmacists in this study believed their skills could be utilised more effectively to encourage vaccine uptake, but they felt that additional support would be required from national bodies to enable this. Practice nurses in this study were familiar with all vaccinations on the current immunisation schedule and regularly engaged in patient consultations to discuss vaccines. Similar to pharmacists, they believed that developing long standing relationships with their patients was important for their recommendations to be accepted. GPs in this study were involved in the delivery of immunisation services but did not regard it as being a major part of their practice. The role of administering vaccines was largely delegated to the practice nurse. Like the other HCPs in this study, they believed it was important not to force the subject, and that their role was to facilitate the patient to make an informed decision. Many GPs were reluctant to “waste” too much time talking about vaccines if they believed the patient’s views could not be changed. In the case of patients who were already open to vaccination, participants believed little discussion or information provision was required.

## Discussion

Using a qualitative, multidisciplinary approach this research has found that the vaccines communication process between HCP and patient is multifactorial and relies greatly on the perceptions of the HCP and their relationship with a specific patient. A fundamental finding of this study was the recognition by HCPs of the important role they play in vaccination services. They acknowledged that they have a responsibility to educate, and advocate for vaccination, and they often adopt the position of role model in doing this.

### Comparison with previous research

#### Building a relationship

The HCPs interviewed used what they learned in their experience with disease and with the public to build a relationship with their patients, in the hope of earning their trust and encouraging vaccines uptake. HCPs were emotionally invested, consistent with previous research, and often expressed frustration when faced with resistance [32]. This study found, like many others, that the motivation behind information provision is a sense of duty of care for the community, patient [4] and the requirement for informed consent and respecting patient autonomy [13, 14, 21–28, 41, 42]. Participants felt it was important to maintain the clinical rapport rather than engage in conflict with vaccine hesitant patients, similar to other studies [13, 28, 32, 43]. In the midst of the ongoing COVID-19 pandemic, the subject of vaccines remain as emotionally charged as ever. It has been suggested that effective education strategies should account for the emotional state of the individual, and that this knowledge can be used to address vaccine hesitancy and communicate information more effectively [44].

#### Perception of risk

The literature supports our participants’ views that the public often perceive the risk of disease or VPD as being low, and the risk of adverse effects of vaccination as being high, therein creating significant barriers to vaccine uptake [45–48]. Patients who relied on HCPs as a credible information source, were more likely to associate vaccines with effectiveness [10]. Additionally, HCPs in our study believed the public do not fear disease in the same way, and that they do not care about herd immunity, but are driven by a desire to protect themselves and their family. HCPs in this study did not regard herd immunity as a worthwhile topic to discuss with patients. However, in light of the COVID-19 pandemic, there has been a greater emphasis on how an individual’s actions can affect the community. Herd immunity is garnering a greater understanding and people may want to adopt preventative measures to fulfil their social obligations [49], e.g. wearing masks or practicing hand hygiene.

#### Trust

Participants reported that patients would often defer the decision to their trusted HCP, similar to findings of several studies [25, 50]. A systematic review found that trust in governments and HCPs correlated positively with an increased intention to vaccinate [30]. Another review revealed that distrust of doctors, government sources and pharmaceutical companies are the main reasons for vaccine hesitancy [4]. HCPs in this study expressed concern over the possibility of inconsistent information from HCPs damaging public trust in the medical community.

#### Preconceived views

Our participants perceived that patients have already decided whether or not to vaccinate before speaking with a HCP. Consequently, some HCPs adopted a passive approach to the topic, whereby the issue was only discussed in reaction to questions from patients. A similar finding was reported in other studies where factors such as ambiguity over roles, environmental constraints, and concerns over necessity or safety prevented HCPs from actively broaching the subject [17–21, 26, 31]. In our study, discussions about childhood vaccines were routinely initiated at the six-week check, otherwise, much discussion occurred in an ad hoc manner. There may be missed opportunities to discuss vaccinations with patients as a result.

### Implications for future research and practice

This study contributes to the developing body of evidence to inform the development of effective communication strategies to increase vaccine uptake [25, 27, 28, 32–34, 51, 52]. Research has established the importance of communication in addressing vaccine hesitancy [53]. An evidence-based method, with consistent and national implementation by all HCPs e.g. the Communicate to Vaccinate (COMMVAC) method, could guide future initiatives to address vaccine uptake [54]. An opportunity for proactive, consistent multidisciplinary vaccines communication training exists to bolster a unified HCP approach.

A limitation of the study is the small sample size and that all interviews were conducted in one region in Ireland. Selection bias may lead to the views of those willing to be interviewed being captured. However, this is presented clearly and the inclusion of participants from different healthcare professional groups increases the representation of the findings. Similarly, the prevalence and consistency of findings, is outlined in the results.

## Conclusions

This study adds to the small body of research investigating HCP views and experiences of providing vaccines information to their patients. It was identified that HCP-patient relationship, trust and perception of risk are important factors to address to encourage vaccine uptake. Given the growing importance of promoting vaccines uptake in light of the decreasing uptake of certain vaccines and the COVID-19 pandemic, support and evidence-based interventions for HCPs in communicating vaccines information to their patients is recommended.

## Supplementary Information

Below is the link to the electronic supplementary material.

Supplementary file 1 (DOCX 13 kb)

Supplementary file 2 (DOCX 16 kb)

## Data Availability

Data is not and will not be made available elsewhere.
