# Supplementary material for: A qualitative study of the views of healthcare professionals on providing vaccines information to patients
Source: Int J Clin Pharm. 2021 Jun 21;43(6):1683–92. doi: 10.1007/s11096-021-01299-y (PMC8216584; doi:10.1007/s11096-021-01299-y)
Supplement: Supplementary file 1 — (DOCX 13 kb) [file 11096_2021_1299_MOESM1_ESM.docx]

Topic Guide

Views/perceptions/experiences of HCPs

How do you view your role in the provision of vaccines services?

How much of your time is spent speaking to people /providing information about vaccines?

Are there vaccines that you are more likely to recommend than others?

Which vaccines do you feel are most important for people to receive?

Why do you feel these vaccines are more important?

What concerns do you have regarding the safety of certain vaccines?

Are there groups of patients that you are more likely to recommend certain vaccines to?

Do you think there are some recommended vaccines that are not recommended?

DO you think there are some recommended vaccines that are not necessary?

How do you feel about the current immunisation recommendations in Ireland?

How familiar are you with it?

What are your experiences of the public’s perception of vaccines?

Where do you think people get their vaccines information?

Are there variations among groups of patients in their attitudes towards vaccines?

What do you think are the main barriers and facilitators to availing of vaccination services?

Providing information by HCPs

Can you tell me about your experience of discussing vaccines with patients?

When discussing vaccines what does the conversation usually centre on?

Do you bring it up/does the patient bring it up?

DO you feel the topic is important to discuss and why?

How do you address concerns from patients regarding the safety of vaccines?

What are the main concerns that you are faced with?

What kind of information formats do you find helpful in addressing concerns?

What kind of information do you think would help you if it were available?

How confident do you feel about the effectiveness of various vaccines?

What kind of information do you seek in assessing the efficacy of vaccines?

What resources do you use to find information regarding a vaccine?

Do you have to proactively seek out vaccines information or is information circulated to you?
