# Supplementary material for: A qualitative study of the views of healthcare professionals on providing vaccines information to patients
Source: Int J Clin Pharm. 2021 Jun 21;43(6):1683–92. doi: 10.1007/s11096-021-01299-y (PMC8216584; doi:10.1007/s11096-021-01299-y)
Supplement: Supplementary file 2 — (DOCX 16 kb) [file 11096_2021_1299_MOESM2_ESM.docx]

COREQ (COnsolidated criteria for REporting Qualitative research) Checklist.

| **Domain 1: Research team and reflexivity** |  |
| --- | --- |
| 1. Interviewer | Author Ruth Loftus |
| 1. Credentials | MPharm, MPSI |
| 1. Occupation | Pharmacist, clinical researcher |
| 1. Gender | Female |
| 1. Experience and training | Experience as a clinical pharmacist, trained in qualitative research methods |
| Relationship with participants |  |
| 1. Relationship established | Researcher was known to some participants in a professional capacity |
| 1. Participant knowledge of interviewer | Participants were aware that RL was a pharmacist and conducting this research for a Masters dissertation |
| 1. Interviewer characteristics | Pharmacist and clinical pharmacy researcher, no bias or assumptions present to report |
| **Domain 2: Study design** |  |
| *Theoretical framework* |  |
| 1. Methodological orientation and theory | Semi-structured interviews, thematic analysis |
| *Participant selection* |  |
| 1. Sampling | Convenience sampling |
| 1. Method of approach | Telephone contact |
| 1. Sample size | 14 |
| 1. Non participation | 4 people invited declined to participate due to lack of time. No participants dropped out. |
| 1. Setting of data collection | All interviews were conducted at the participants work location e.g. general practice clinic office or pharmacy, in a private room, without disruption. |
| 1. Presence of non-participants | No. |
| 1. Description of samples | 6 participants were female and 8 participants were male. The interviews took place between April and August 2019. |
| *Data collection* |  |
| 1. Interview guide | A topic guide was developed based on a review of the literature and research team experience in the area of vaccinations and vaccine hesitancy. The topic guide was piloted with one participant and this interview was included in the study. |
| 1. Repeat interviews | No. |
| 1. Audio/visual recording | 13 interviews were audio-recorded, one participant declined audio-recording and RL took detailed notes during and field notes after the interview. |
| 1. Field notes | Field notes were recorded after each interview. |
| 1. Duration | The mean interview length was 28 minutes and ranged from 19 to 66 minutes. |
| 1. Data saturation | Data analysis coincided with data collection. It was agreed by the authors that if no new themes emerged in the additional three interviews after the eleventh interview (i.e. 14 interviews in total) then this would confirm that data saturation had been reached. |
| 1. Transcripts returned | No |
| **Domain 3: Analysis and findings** |  |
| *Data analysis* |  |
| 1. Number of data coders | All three authors were involved in data coding. |
| 1. Description of the coding tree | Transcripts were analysed by inductive thematic analysis, with constant comparison methods applied throughout. |
| 1. Derivation of themes | Conventional thematic analysis was conducted to create initial, non-hierarchical odes which were then reviewed and themes and subthemes were developed. |
| 1. Software | No |
| 1. Participant checking | Participants did not provide feedback on findings |
| *Reporting* |  |
| 1. Quotations presented | Quotations are presented |
| 1. Data and findings consistent | There was consistency between the findings and the data presented. |
| 1. Clarity of major themes | Major themes are clearly presented in the results section. |
| 1. Clarity of minor themes | Subthemes are presented under each of the major themes. |
